# A life prediction method based on MDFF and DITCN-ABiGRU mixed network model

**DOI:** 10.1016/j.heliyon.2024.e24299

**Published:** 2024-01-09

**Authors:** Weixiao Xu, Yujie Shen, Luyang Jing, Xianbin Sun

**Affiliations:** Qingdao Technological University, Shandong Qingdao, 266520, China

**Keywords:** Temporal convolutional network, Multi-domain feature fusion, Rotating machinery, Life prediction

## Abstract

A single network model exhibits limitations in the life prediction of rotating machinery for the various fault types and uncertain fault occurrence. Therefore, a network prediction model combining multi-domain feature fusion (MDFF) and distributed TCN-Attention-BiGRU (DITCN-ABiGRU) is proposed to enable a more accurate life prediction of rotating machinery. Firstly, the features of vibration signals collected from multiple sensors are extracted in the time, frequency, and time-frequency domains. Subsequently, dimensionality reduction optimization is conducted on these multi-domain features to eliminate useless information features. The temporal convolutional network (TCN) model is constructed to capture the critical information reflecting the fault characteristics of rotating machinery through the attention mechanism, and the dependencies of the whole training process are captured by the BiGRU network. Finally, precise prediction of the lifespan of rotating machinery is achieved by constructing a health indicator curve (HI). The proposed methods are verified through the life prediction of rolling bearings from the IEEE PHM Challenge 2012 dataset and ball screw pairs from a designed experiment. The experimental results show that the proposed MDFF and DITCN-ABiGRU model achieves a better score and lower error than the convolutional neural network (CNN) and GRU models.

## 1 Introduction

In recent years, there has been a growing trend towards increased automation and intelligence in rotating machinery, accompanied by more stringent requirements for accuracy and safety. Operating under various extreme conditions including temperature, humidity, and vibration over extended periods, rotating machinery is susceptible to performance degradation and reduced residual life. The failure of even a single component within the rotating machinery can lead to the failure of the entire equipment or even the entire system, thereby impacting both enterprise productivity and human safety. Among these vital components, rolling bearings, ball screws, and other crucial parts of rotating machinery are particularly prone to failure [[1], [2], [3]].

To comprehensively and accurately describe the performance degradation trend of rotating machinery, this study focuses on rolling bearings and ball screws as the research objects for investigating the life prediction of key mechanical components [4,5]. This approach aims to mitigate the risks associated with the use of rotating machinery equipment, reduce maintenance costs for enterprises, and enhance overall economic benefits.

Deep learning theory-based life prediction methods have gained significant popularity in predicting the lifespan of mechanical equipment due to their ability to extract meaningful features from vast amounts of data without relying on explicit physical models. Various deep learning models have been widely employed, including Convolutional Neural Networks (CNN), Deep Autoencoder (DAE), Long Short-Term Memory (LSTM), Gated Recurrent Unit (GRU), and Temporal Convolutional Network (TCN). For instance, Li et al. [6] utilized recurrent neural networks coupled with reinforcement learning units to predict the state trend of rolling bearings. Deutsch et al. [7] combined big data computing methods with machine learning techniques and proposed a novel residual life prediction method based on Deep Belief Network (DBN), demonstrating its effectiveness through verification experiments. Yoo et al. [8] applied continuous wavelet transform and convolutional neural network models to forecast the remaining service life of equipment.

The application of deep learning methods in predicting the lifespan of rotating machinery has shown promising outcomes. However, given the diverse range of faults encountered during the operation of rotating machinery, which inherently entail uncertainties, the utilization of a single network model for life prediction presents certain limitations. For instance, traditional CNN models may encounter challenges such as vanishing or exploding gradients when applied to time series data prediction. RNN and LSTM network models are susceptible to difficulties in capturing long-range dependencies when handling extensive time series data [9]. Similarly, a standalone TCN model may struggle to accurately capture sensitive and interconnected information, and may exhibit limited robustness. To address these issues, researchers and experts have adopted a hybrid model approach for life prediction, enhancing the original models by integrating and leveraging the strengths of each model. This strategy aims to achieve accurate life prediction of rotating machinery by effectively combining multiple models.

Kong et al. [10] employed polynomial regression, CNN, and LSTM to construct a health index (HI) for predicting the Remaining Useful Life (RUL) of the device. An et al. [11] integrated CNN with stacked LSTM networks to forecast the remaining life of milling cutters. Xu et al. [12] proposed a novel degradation-trend-constrained VAE (DTC-VAE) to construct a distinct degradation-trend HI vector, yielding favorable prediction results. Li et al. [13] explored a dual-thread gated recurrent unit (DTGRU) to enhance predictive capability for complex degradation trajectories. This approach incorporated a dual-thread learning strategy to capture stationary and nonstationary information from input data and the difference in hidden states between adjacent time steps. Zhang et al. [14] introduced the cocktail of long short-term memory (C-LSTM), a novel multihierarchy network based on multiordered neurons, for accurate long-term RUL prediction of gearboxes and bearings. Ding et al. [15] obtained cross-domain characteristics of bearings by domain adaptive (DA), and proposed a prediction method of rolling bearing RUL based on depth transfer autoencoder. Cao et al. [16] proposed the use of the TCN model for systematic evaluation of general convolutional and cyclic architectures for serial modeling, resulting in improved prediction performance. Thus, by constructing a hybrid life prediction model, various prediction models can be concatenated to leverage their respective advantages and achieve accurate life prediction of rotating machinery.

In this paper, we present a life prediction model based on the DITCN-ABiGRU hybrid network. Our approach involves extracting multiple sensitive features that reflect the operational status of the ball screw pair, which are then used to construct a health index (HI). Additionally, we introduce a TCN network to create a multi-domain feature fusion multi-convolutional kernel parallel network. To enhance the prediction accuracy of the degradation trend, we incorporate an attention mechanism to highlight the features that are sensitive to the degradation trend. Experimental data from ball screw pairs in the laboratory and the IEEE PHM Challenge 2012 bearing open data sets are utilized to validate the effectiveness and reliability of our proposed method.

## 2 Related works

### 2.1 Temporal convolutional network

TCN is a temporal model for processing temporal data composed of causal, extended, and residual link modules [[17], [18], [19]]. It is a variant of CNN, which has the advantage of predicting future information based on past information without information leakage, and can take any length of sequence as input and map it to the output sequence of the same length. In the actual data processing process, causal convolution is used to process temporal data, and extended convolution is used to deal with the common long-distance dependence problem in temporal models. Its parallel structure is more conducive to processing temporal sequence-long data.

The network model of the TCN can be described as follows in (1). For a given input sequence, there exists a prediction function such that the input sequence corresponds to an output sequence.

(1) $$y_{t}=f\left(x_{1},x_{2}\cdots x_{t}\right),t=1,2,\cdots T$$

(1) Causal Convolution and Dilated Convolution

**Fig. 1:**
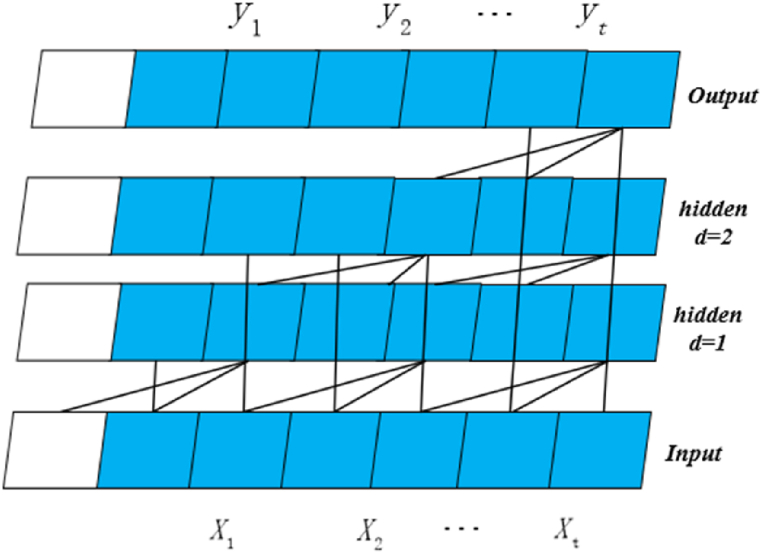
Schematic diagram of dilated convolution structure.

Causal convolution can help the network to remember past information, which is prone to the problem that the amount of information is too large and the efficiency of the training model is reduced. Therefore, it is necessary to introduce expansive convolution, where the structure of expansive convolution is shown in Fig. 1. The dilated convolution can be displayed as (2).

(2) $$F\left(x\right)=\left(x*f_{d}\right)\left(x\sum_{i=0}^{n-1}f\left(.\right)*x_{x-di}\right)$$

Where, d is the inflation factor, n is the filter size, and $x_{x-\text{di}}$ represents the sequence elements multiplied by the elements in the convolution kernel. The filter in dilated convolution obtains information further from the current input by skipping some input values and generally takes the exponent of 2 (1,2,4,8 …,2 m) as the expansion rate.

(2) Residual Connections

To solve the problems of gradient disappearance, gradient explosion, and network performance degradation in the deep network, a residual connection module is introduced in TCN to replace the convolutional layer. Non-adjacent layers can carry out information transmission and input information is weighted, and fused into the output model [20].

**Fig. 2:**
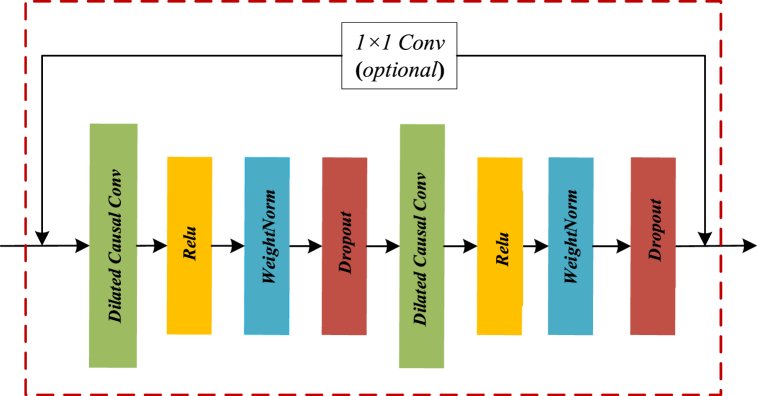
Residual connections.

The residual structure of the temporal CNN is shown in the following Fig. 2. The input of the model is weighted and fused into the output of the model to obtain the final TCN output, which is specifically expressed explicitly in (3).

(3) $$y=G\left(x,\left\{w_{i}\right\}\right)+x$$

### 2.2 Bidirectional gate recurrent unit

The Gate Recurrent Unit (GRU) model can better capture the dependency relationships with large intervals in timing data, and it can effectively solve problems such as long-term memory and gradient in backpropagation [21,22], thus simplifying the structure of the LSTM network on the premise of maintaining neuronal memory. Train your speed in advance.

**Fig. 3:**
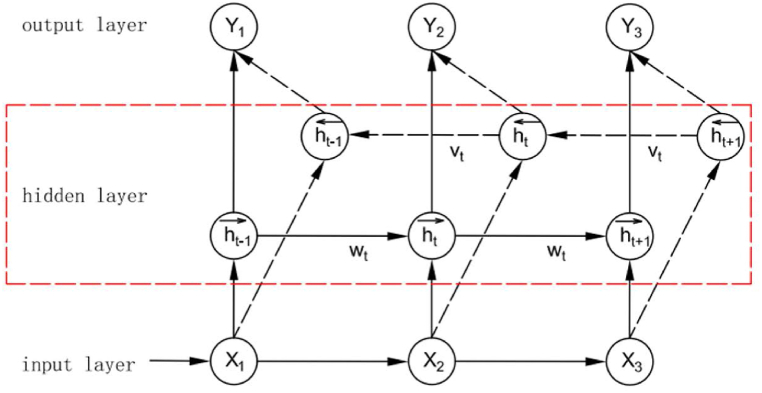
Structure of BiGRU network model.

For the time series prediction problem of rotating machinery, the GRU network only considers the influence of the past time series data characteristics of rotating machinery on the following time series data characteristics. It does not consider the correlation factors of the previous time characteristics and the next time information characteristics. Therefore, this paper uses the BiGRU network model to realize the learning of both the historical time input feature data and the current input feature data and merge the future feature data information [23]. The structure of the BiGRU network model is shown in Fig. 3.

### 2.3 Global attention

When processing a large amount of data, the global attention mechanism is added to change the weight of key features in the TCN network and screen out important feature information, to strengthen the influence of important features on the degradation trend and improve the accuracy of prediction [[24], [25], [26]]. The attention weight vector is generated by updating the state information at s time of the encoder layer and the target state information at the top layer of the decoder layer. The calculation formulas of attention weight vector, context vector and target state information are shown in (4), (5), (6), (7).

(4) $$x_{t}=h_{s}^{T}h_{t}$$

(5) $$a_{t}=\frac{\exp\left(x_{t}\right)}{\sum_{j=0}^{t}\;\exp\left(x_{j}\right)}$$

(6) $$y_{t}=softmax\left(W_{s}\tilde{h_{t}}\right)$$

(7) $$c_{t}=\sum_{t=0}^{T}{a_{t}h}_{t}$$

Where, $W_{s}$ is the weight matrix of global attention training.

### 2.4 Construct evaluation indicators

To compare the effects of different prediction models, two indices, error, and score, are used to measure the prediction results of each model. The calculation formula of the $E_{i},A_{i}$ and Score are shown in (8), (9), (10).

(8) $$E_{i}=\frac{{actRUL}_{i}-{preRUL}_{i}}{{actRUL}_{i}}\times 100\%$$

(9) $$A_{i}=\left\{ \begin{array}{c} \exp^{-\ln\;\left(0.5\right)∙\left(E_{i}/5\right)},E_{i}\leq 0\\ \exp^{\ln\;\left(0.5\right)∙\left(E_{i}/20\right)},E_{i}>0 \end{array}\right.$$

(10) $$\text{Score}=\frac{1}{n}\sum_{i=1}^{n}A_{i}$$

**Fig. 4:**
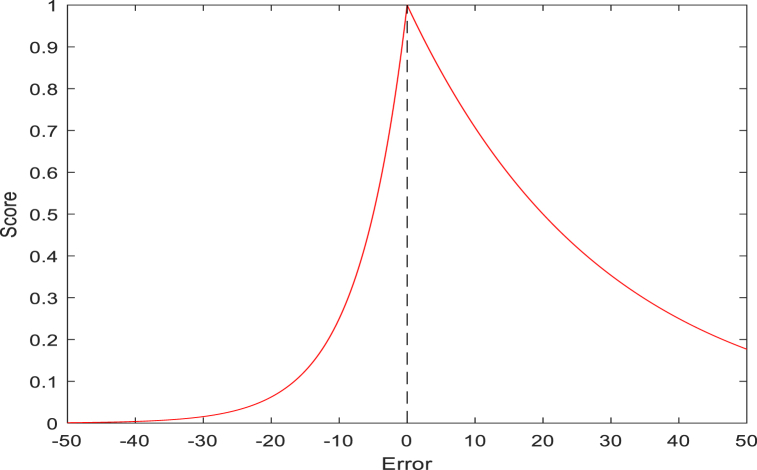
Score vs the corresponding percent error.

As can be seen from Fig. 4, the function relationship between score and error, the value of error is between −50 and 50. When the error was 0, the prediction error was 0, the score was 1, and the positive error had a higher score than the negative error. It can be observed that the underestimation of the life prediction of rotating machinery is more meaningful for life prediction.

## 3 Innovative works

### 3.1 The model based on MDFF and DITCN-ABiGRU

In this paper, a DITCN-ABiGRU network prediction model is proposed based on the advantages of the TCN network, which can perform convolution in parallel, has more extended memory, flexible receptive field, and strong time feature extraction ability of BiGRU. The advantages of the two models are that they are to realize the deep extraction of spatiotemporal relationship features, making them more suitable for the life prediction of rotating machinery.

**Fig. 5:**
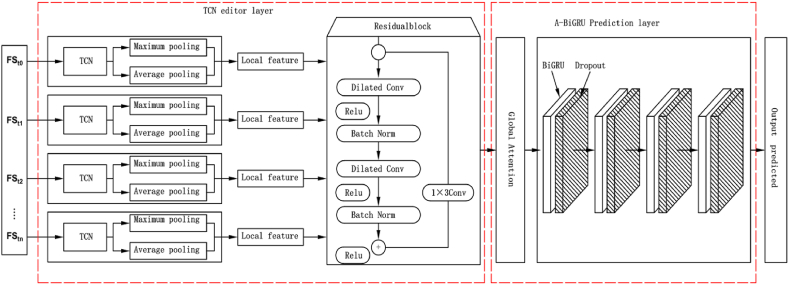
The network model based on DITCN-ABiGRU.

The model has the following two innovation points: Innovation point 1: Because different features have different sensitivities to other degradation states, the TCN network can be used to perform convolution in parallel, and the convolution kernel of various sizes can be used to achieve parallel extraction of multi-scale features, which can better retain the information of each layer in the middle and obtain a faster convergence rate. Innovation point 2: The attention mechanism is introduced to calculate the importance degree of different features, so as to improve the accuracy of life prediction. The bidirectional gated recurrent unit is introduced to further fuse the sensitive feature quantities and classify them, and a faster convergence speed is obtained in Fig. 5, which shows the network model based on DITCN-ABiGRU.

### 3.2 Network prediction process based on MDFF and DITCN-ABiGRU

**Fig. 6:**
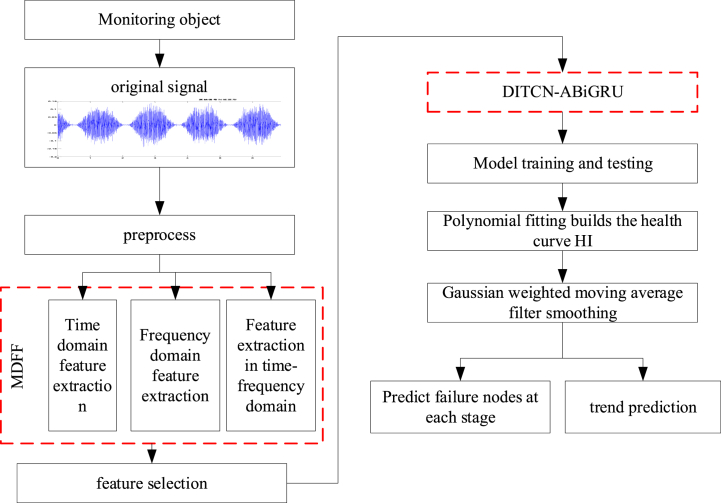
Flow chart of network prediction based on MDFF and DITCN-ABiGRU.

The network prediction flow chart based on MDFF and DITCN-ABiGRU model is shown in Fig. 6. The model is mainly composed of the following parts.

Step 1 Multi-domain feature extraction and optimization. After the vibration signals are preprocessed, the time domain and frequency domain features are extracted respectively, and the multi-domain feature quantity fusion is realized.

Step 2 Data processing and screening. The random forest algorithm is used to reduce the dimensionality of the extracted multi-domain feature quantity and screen out the sensitive feature quantity.

Step 3 Build the DITCN-ABiGRU network model. The number of TCN residual units and BiGRU network layers is adjusted according to the number of features, and the parameters of the network model, such as the activation function used, are determined.

Step 4 An attention mechanism was introduced to construct the health factor curve HI of the rotating machinery.

Step 5 Model training: the HI output of the model is smoothed by a Gaussian filter.

Step 6 The failure time node of the degradation stage is calculated using polynomial fitting. The calculation formula is as follows in(11).

(11) $$G\left(x\right)=\frac{1}{\sqrt{2\pi }\sigma }e^{-\frac{x^{2}}{2\sigma^{2}}}$$

where, X represents the lead screw prediction HI and G(x) is the lead screw prediction HI after Gaussian filtering.

Step 7 Comparative analysis using an evaluation index for error assessment.

## 4 Experiment and discussion

To verify the feasibility of the method proposed in this paper, the IEEE PHM Challenge 2012 bearing a public dataset [27] and the existing experimental data of ball screw subs in the laboratory are used to predict the life of rolling bearings and ball screw subs, respectively.

### 4.1 Life prediction of ball screw pair

#### 4.1.1 Experimental setup

**Fig. 7:**
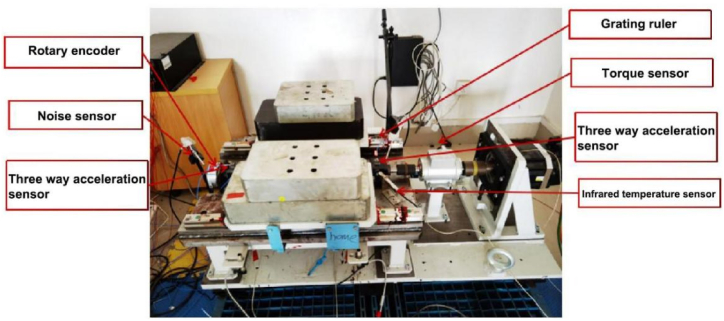
Existing ball screw sub test bench in the laboratory.

A test was conducted on a ball screw test bench designed by the existing laboratory itself, as shown in Fig. 7. The vibration signals at the motor end, bearing seat end, and fillet vice are collected by the three-way acceleration sensors to monitor the full life of the ball screw vice, with a sampling frequency of 10 khz, once every 3 h, which is a total of nine vibration sensors, comprising a total of 9 × 3314 groups of multi-source information.

#### 4.1.2 Experimental data processing

**Fig. 8:**
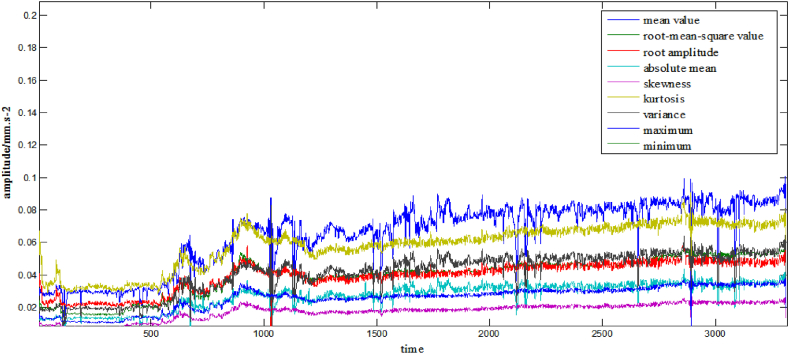
Ball screw sub feature value display.

Extract the feature quantity of the original signal of the ball screw pair. To maximize the life prediction of the ball screw sub, 15-time domain eigenvalues (root mean square value, root amplitude, absolute mean value and other time domain indexes), four frequency domain eigenvalues (center of gravity frequency, root mean honest frequency, and other four frequency domain indexes), and four time-frequency domain eigenvalues are manually extracted from each pre-processed original signal, respectively, to form a total of 23 fusion feature quantities. The ball screw sub-feature value display is shown in Fig. 8.

After normalization, the fused multi-domain feature set is input into the random forest, and the RF algorithm is used to screen the variables of the feature quantities that affect the fault state of the ball screw pair to obtain the sensitive feature quantities. Finally, 15 liable feature quantities that have an essential impact on the ball screw pair system are selected for life prediction.

#### 4.1.3 Network structure setup

In this model, the activation function of the residual block of the TCN is leaky rectified linear units (Leaky Re LU), where the random loss rate of dropout is 0.5, and the data after the convolution operation is normalized by layer normalization; thus, the mean value of the input data of the Leaky Re LU layer is 0 and the variance is 1. The number of neurons in the fully connected layer was 16, the output layer was 1, the activation function of the output layer was Re LU, the learning rate was 0.001, and the number of training sessions was 80.

#### 4.1.4 Analysis of experimental results

**Fig. 9:**
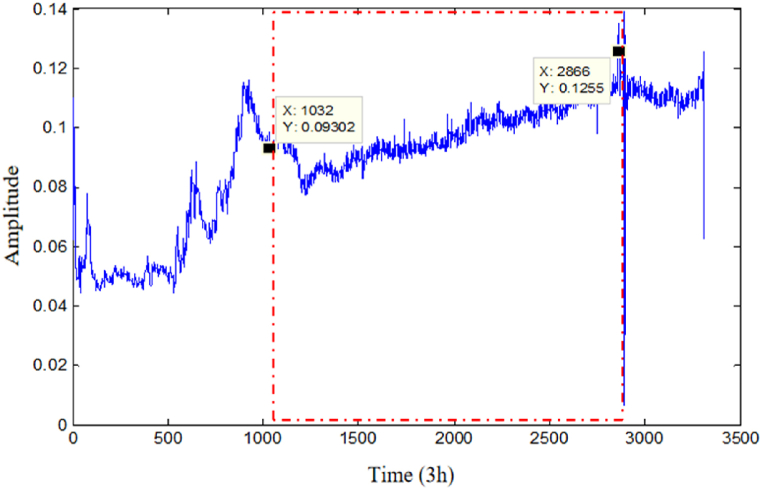
Ball screw sub degradation stage division.

The 3314-time series of the vibration signal reflecting the state of the ball screw sub from normal to failure during the whole life cycle are processed. By processing the fused feature information, it is known that the grinding period ends at 1034 (3102 h) and the screw sub enters a stable wear period, and the smooth wear period ends at 2866 (8598 h) and enters a severe wear stage, and the specific degradation stage curve is shown in Fig. 9. The first 80 % time-series samples of each degradation stage are selected as the test set to predict the failure time point at the end of each degradation stage. Table 1 shows the parameter setting of DITCN-ABiGRU model.

**Table 1:** The parameter setting of DITCN-ABiGRU model.

Model	Parameter
Activation function of TCN	Leaky Re LU
Dropout	0.5
Expansion rate	1、2、4、8
Neurons number of BiGRU	300、256、128
Fully connected layer	16
Output layer activation function	Relu
Learning rate	0.001
Training times	100

**Fig. 10:**
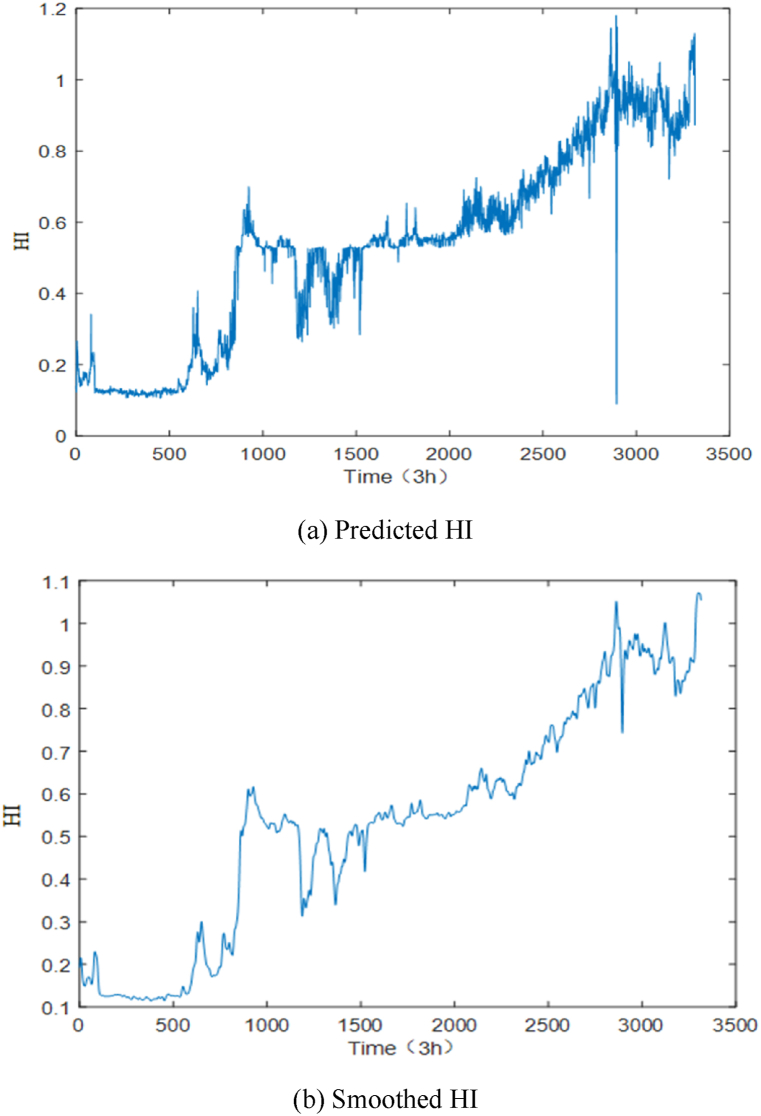
HI curve comparison chart.

The data are input to the trained network model for testing, and the HI curve of the ball screw sub is input and smoothed by a Gaussian filter. The obtained health indicators are smoothed, and the HI curve smoothed by Gaussian filtering is shown in Fig. 10.

To verify the effectiveness of the proposed method, two sets of comparative tests are set up.

(1) Comparison of running time of different models

To compare the effectiveness of the model proposed in this paper, the number of iterations for each model is set to 100, and the training batch is set to 16. Then the running time of CNN, GRU, TCN and the model proposed in this paper is shown in Table 2.

(2) Comparison of evaluation indexes of different models

**Table 2:** The running time of different models.

Models	CNN	GRU	TCN	DITCN-ABiGRU
run time(s)	4.17	4.62	4.11	2.36

To reflect the method's effectiveness in this study, CNN, GRU, and TCN network models are used for the comparison experiments. To facilitate comparison, this experiment used $E_{i}$ and score as evaluation indexes, and the final experimental results are shown in Table 3.

(1) The error values in the prediction results using the DITCN-ABiGRU network model are lower in the prediction of the three stages of failure points, with higher score values, which are better than those of the CNN, and GRU network models, and the predicted failure time nodes are closer to the actual values. Meanwhile, DITCN-ABiGRU is more favorable for assigning the weights of the model, and its prediction results are more stable.
(2) In the prediction of health index HI, the score of the DITCN-ABiGRU network model is 0.5567, which is 38.54 %, 14.24 % and 6.88 % higher than that of CNN, GRU, and TCN network models, respectively. It shows that the method proposed in this paper can improve the accuracy of life prediction under variable working conditions.

**Table 3:** Comparison of test results of each model.

Predicting failure time points		Run-in period	Stable period	Catagen period	Score
Actual expiration time node (3 h)		1032	2866	3314	–
Predict RUL (3 h)	CNN	1486	2541	3564	–
	GRU	1436	2745	3451	–
	TCN	1417	2893	3409	–
	DITCN-ABiGRU	1303	2824	3400	–
$E_{i}$	CNN	−43.99	11.34	−7.54	0.3429
	GRU	−39.15	4.22	−4.13	0.4774
	TCN	−27.17	−0.94	−2.87	0.5184
	DITCN-ABiGRU	−26.26	1.61	−2.60	0.5567

**Fig. 11:**
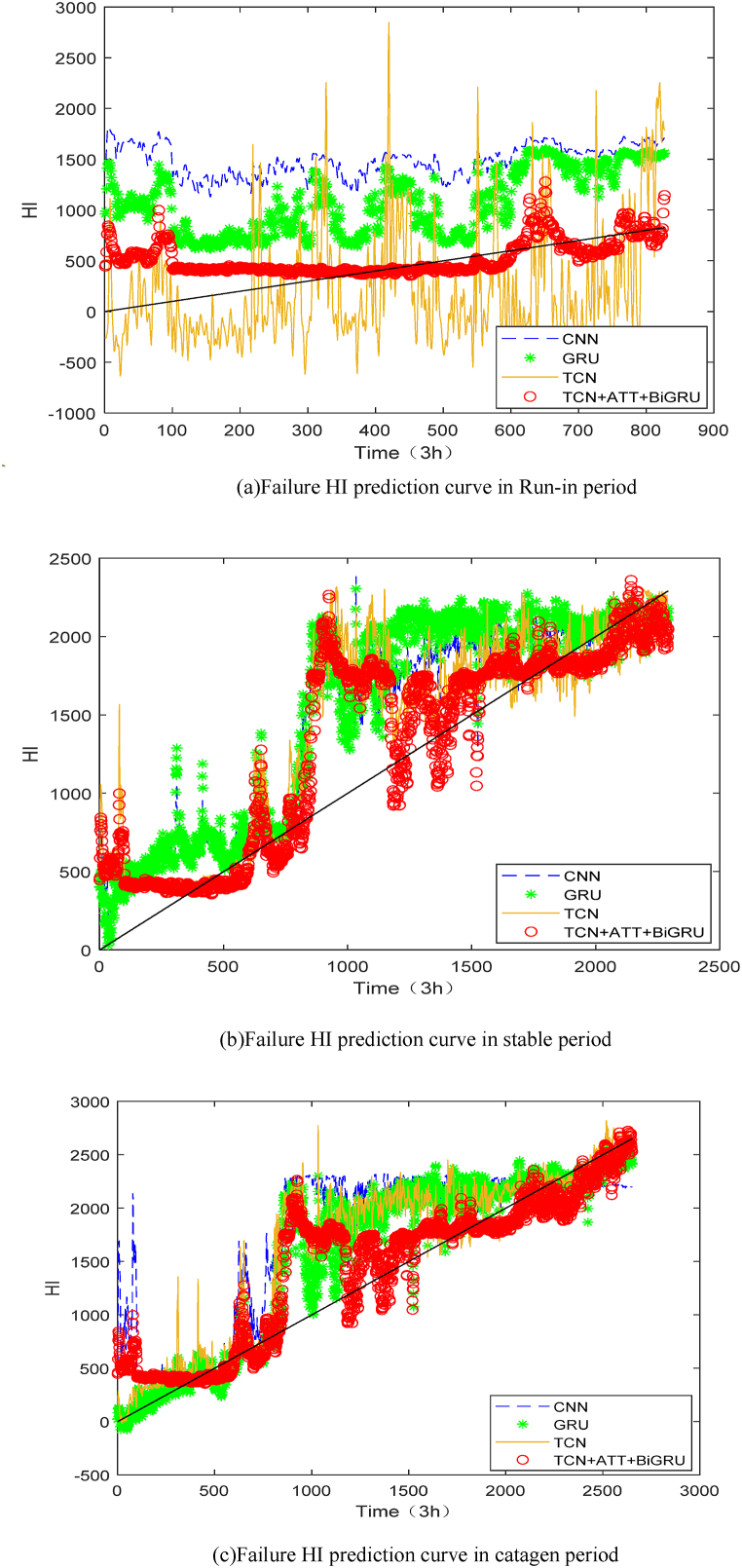
Comparison of failure HI prediction curves of different models.

Based on Table 3 and Fig. 11, and the comparison among different models, the following conclusions can be drawn.

### 4.2 Life prediction of rolling bearings

#### 4.2.1 Experimental setup

**Fig. 12:**
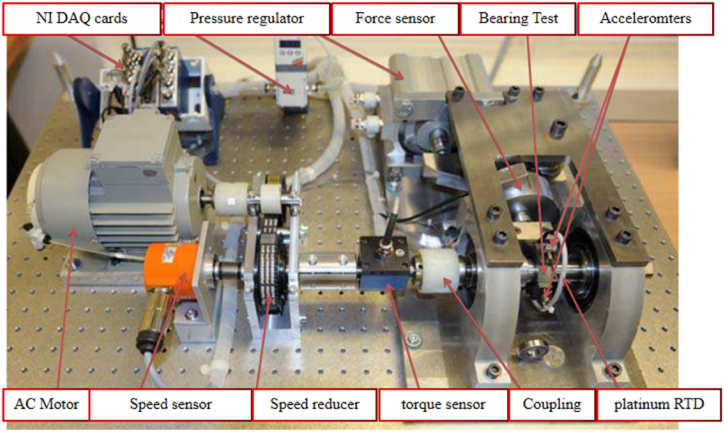
Composition of the PRONOSTIA experimental platform.

The PRONOSTIA experimental platform consists of a rotating part, load part, and data acquisition part, as shown in Fig. 12. The vibration signals are collected by accelerometers placed in horizontal and vertical directions with a sampling frequency of 25.6 kHz, recorded at 10 s intervals, with a duration of 0.1 s for each acquisition, and 2560 data points for each time. The operating conditions of the PRONOSTIA experimental platform are shown in Table 4.

**Table 4:** Operating conditions of the PRONOSTIA experimental platform.

Working condition	condition 1	condition 2	condition 3
Motor speed	800 r/min	650 r/min	1500 r/min
Load	4000 N	4200 N	5000 N

In this paper, the vibration data of bearing 1-1, bearing 1–2, bearing 2–1, bearing 2-2, bearing 3–1 and bearing 3–2 are selected as the training set under three different working conditions, and the vibration data of bearings 1–3, 1–4, 1–5, 1–6, 1–7, 2–3, 2–4, 2–5, 2–6, 2–7 and 3-3, totaling 11 bearings, are selected as the test set.

#### 4.2.2 Data processing

**Fig. 13:**
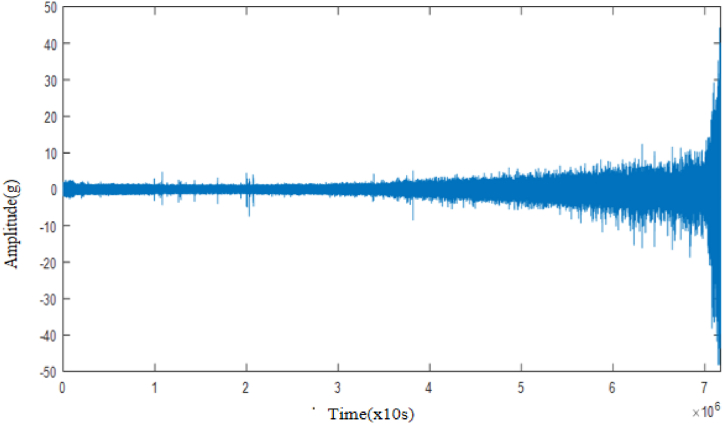
Raw vibration signal of rolling bearing for the whole life cycle.

Separate feature extraction of the original signal of the collected rolling bearing, respectively, by extracting 15-time domain eigenvalues such as mean value, root mean square value, square root amplitude and six frequency domain eigenvalues such as frequency amplitude, frequency value and frequency average, as well as after wavelet packet decomposition, extracting one low-frequency energy value of the third layer, constituting a total of 22 eigenvalues, of which the original vibration signal of the rolling bearing full life cycle is shown in Fig. 13.

#### 4.2.3 Analysis of experimental results

To verify the superiority of the prediction model proposed in this paper, it is compared with the prediction model in the literature. The Introduction to the experimental data set is shown in Table 5.

**Table 5:** Introduction to the experimental data set.

Data set division	Bearing number	Non-full life time ( × 10s)	Full life time ( × 10s)
Training set	1–1	–	2803
	1–2	–	871
	2–1	–	911
	2–2	–	797
	3–1	–	515
	3–2	–	1637
Test set	1–3	1802	2375
	1–4	1139	1428
	1–5	2302	2463
	1–6	2302	2448
	1–7	1502	2259
	2–3	1202	1955
	2–4	612	751
	2–5	2002	2311
	2–6	572	701
	2–7	172	230
	3–3	352	434

In [28], the paper inputs the extracted time domain, frequency domain and time-frequency domain feature sets into RNN to construct health indicators to achieve life prediction. In Ref. [29], the paper extracts local features directly from sensors, and combins convolution with LSTM to predict the life of bearings. In Ref. [30], the paper uses wavelet-EMD decomposition method for feature extraction, and constructed health indicators to achieve life prediction by self-organizing mapping method.

**Fig. 14:**
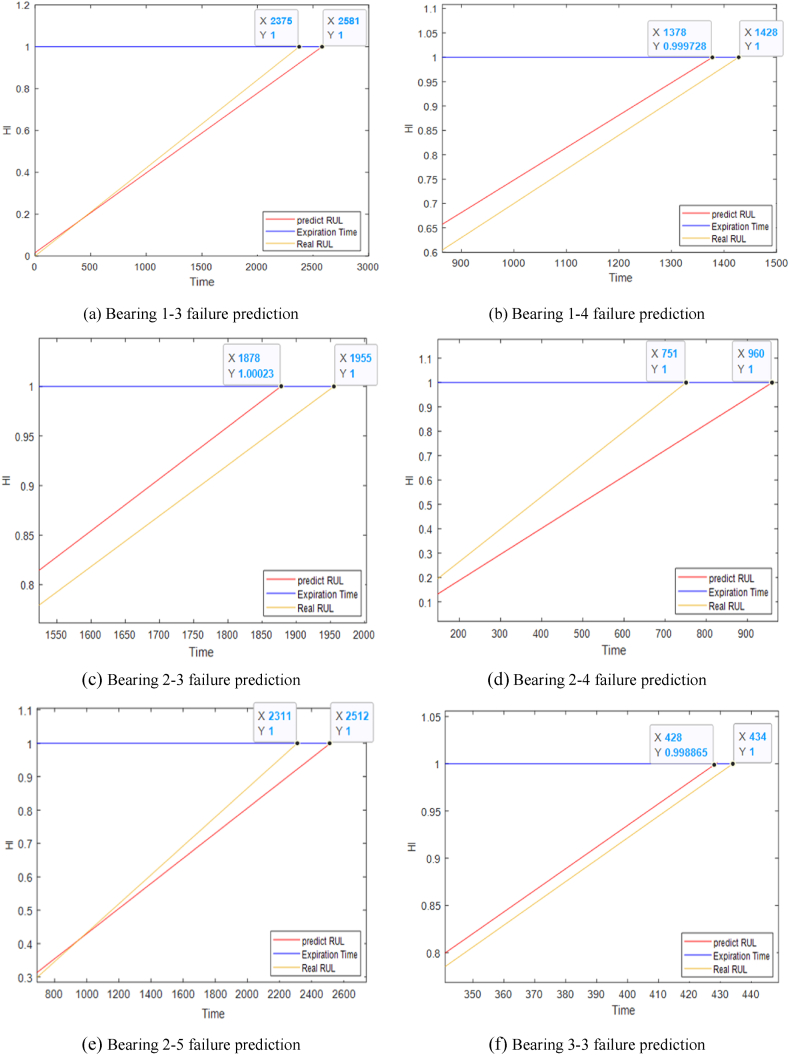
Prediction of partial bearing failure.

The model proposed in this paper is compared with the prediction results of other methods in the literature. Using primary polynomial fitting to calculate the failure time node of the degradation stage of the ball screw sub, the life prediction curve of bearings are shown in Fig. 14.

Through the analysis of Table 6 and Fig. 14, it can be seen that the score value of the prediction model proposed in this paper is 0.4347, which is 40.19 %, 11.87 % and 18.33 % higher than that of References [[28], [29], [30]] respectively. It can be seen that the addition of global attention mechanism and the mixed use of the TCN and BiGRU models can give full play to the advantages of each model and effectively improve the life prediction performance of the model, which contains more useful features than the single performance degradation trend feature. The validity of the model in the life prediction of rolling bearings is verified, which shows that the method proposed in this paper is suitable for life prediction under variable working conditions.

**Table 6:** Comparison and analysis of each model.

Bearing	Real RUL	Predict RUL	$E_{i}$			
			•The paper	Reference [28]	Reference [29]	Reference [30]
Bearing1_3	5730	7790	−35.95	43.28	54.73	−1.04
Bearing1_4	2890	2390	17.30	67.55	38.69	−20.94
Bearing1_5	1610	1720	−6.83	−22.98	−99.4	−278.26
Bearing1_6	1460	1530	−4.79	21.23	−120.07	19.18
Bearing1_7	7570	7680	−1.45	17.83	70.65	−7.13
Bearing2_3	7530	6760	10.23	37.84	75.53	10.49
Bearing2_4	1390	3480	−150.36	−19.42	19.81	51.80
Bearing2_5	3090	5100	−65.05	54.37	8.2	28.80
Bearing2_6	1290	1310	−1.55	−13.95	17.87	−20.93
Bearing2_7	580	670	−15.52	−55.17	1.69	44.83
Bearing3_3	820	790	3.66	3.66	2.93	−3.66
score			0.4347	0.26	0.3828	0.3550

## 5 Conclusion

In this paper, a network prediction model combined with multi-source information fusion and DITCN-ABiGRU was proposed, and the validity of the model was verified using the IEEE PHM Challenge 2012 bearing public dataset and the experimental dataset of ball screw subsets. The results of the study indicated that:

(1) Multi-domain feature fusion can effectively avoid the problem of missing fault information in a single position and can comprehensively monitor the fault information of rotating machinery.
(2) The DITCN-ABiGRU model leverages the advantages of each individual model and significantly improves the life prediction performance of rotating machinery. The TCN network enables parallel convolutions, while the global attention mechanism highlights the feature quantities that are sensitive to the degradation trend, thereby enhancing the prediction accuracy of this trend. Additionally, the bidirectional gated cycle unit integrates the sensitive feature quantities, resulting in faster convergence speed and achieving excellent prediction outcomes.

## Author contributions

Jing Lu-yang: Writing – review & editing, Supervision. Shen Yu-jie: Data curation. Sun Xian-bin: Writing – review & editing. Xu Wei-xiao: Writing – review & editing, Writing – original draft, Data curation

## Data availability statement

The data are not publicly available due to their containing information that could compromise the privacy of research participants.

## Declaration of competing interest

The authors declare that they have no known competing financial interests or personal relationships that could have appeared to influence the work reported in this paper.
